# Long non-coding RNA ZFAS1 interacts with CDK1 and is involved in p53-dependent cell cycle control and apoptosis in colorectal cancer

**DOI:** 10.18632/oncotarget.5807

**Published:** 2015-10-19

**Authors:** Nithyananda Thorenoor, Petra Faltejskova-Vychytilova, Sonja Hombach, Jitka Mlcochova, Markus Kretz, Marek Svoboda, Ondrej Slaby

**Affiliations:** ^1^ Central European Institute of Technology, Masaryk University, 62500 Brno, Czech Republic; ^2^ Masaryk Memorial Cancer Institute, Department of Comprehensive Cancer Care, 65653 Brno, Czech Republic; ^3^ Institute of Biochemistry, Genetics and Microbiology, University of Regensburg, Regensburg, 93053, Germany

**Keywords:** colorectal cancer, lncRNA, ZFAS1, CDK1

## Abstract

We determined expression of 83 long non-coding RNAs (lncRNAs) and identified ZFAS1 to be significantly up-regulated in colorectal cancer (CRC) tissue. In cohort of 119 CRC patients we observed that 111 cases displayed at least two-times higher expression of ZFAS1 in CRC compared to paired normal colorectal tissue (*P* < 0.0001). By use of CRC cell lines (HCT116+/+, HCT116−/− and DLD-1) we showed, that ZFAS1 silencing decreases proliferation through G1-arrest of cell cycle, and also tumorigenicity of CRC cells. We identified Cyclin-dependent kinase 1 (CDK1) as interacting partner of ZFAS1 by pull-down experiment and RNA immunoprecipitation. Further, we have predicted by bioinformatics approach ZFAS1 to sponge miR-590-3p, which was proved to target CDK1. Levels of CDK1 were not affected by ZFAS1 silencing, but cyclin B1 was decreased in both cell lines. We observed significant increase in p53 levels and PARP cleavage in CRC cell lines after ZFAS1 silencing indicating increase in apoptosis. Our data suggest that ZFAS1 may function as oncogene in CRC by two main actions: (i) via destabilization of p53 and through (ii) interaction with CDK1/cyclin B1 complex leading to cell cycle progression and inhibition of apoptosis. However, molecular mechanisms behind these interactions have to be further clarified.

## INTRODUCTION

Colorectal cancer (CRC) is the third most commonly diagnosed cancer in males and the second in females, with over 1.2 million new cases each year [1–3]. Therefore, a substantial amount of studies have investigated molecular abnormalities associated with CRC, in order to learn more about molecular pathogenesis of this disease [4, 5]. Among numerous oncogenes and tumor suppressors demonstrated to be involved in CRC pathogenesis, long non-coding RNAs (lncRNAs) have attracted attentions of many researchers last years for their aberrant expression patterns in colorectal tumor tissue and their associations with clinico-pathological features of CRC. Consequent *in vitro* and *in vivo* analysis indicated also functional role of these lncRNAs (e.g. CCAT2, MALAT1, HOTAIR, GAS5) in the molecular pathology of CRC [6].

LncRNAs are mRNA-like transcripts ranging in length from 200 nucleotides (nt) to ~100 kilobases (kb) yet do not function as templates for protein synthesis. They exhibit cis- or trans-regulatory capabilities, and the mammalian genome encodes > 1000 lncRNAs that have been significantly conserved among mammals [7, 8]. A small number of characterized human lncRNAs have been already associated with diverse biological processes, including epigenetic regulation, alternative splicing, nuclear import, immune surveillance, embryonic stem cell pluripotency, structural components, precursors to small RNAs and regulators of mRNA decay [9–11]. LncRNAs have been also described as competing endogenous RNAs (ceRNAs) or natural microRNAs (miRNAs) sponges having an active role in regulating miRNAs availability within the cell and form intertwined regulatory networks [12, 13]. Moreover, recent reports have implicated lncRNAs as ceRNAs in human diseases including human cancer [14, 15].

LncRNAs are also known to be deregulated under pathological conditions. Dysregulation of lncRNAs expression has been reported not only in CRC but also in different types of cancers including breast cancer, lung cancer, pancreatic cancer, osteosarcoma, hepatocellular carcinoma and leukemia indicating that lncRNA deregulation could be one of the common features of carcinogenesis [16–18], and deregulated lncRNAs therefore may be utilized for cancer diagnosis, prognosis or serve as potential therapeutic targets.

In our study we profiled expression of disease-associated lncRNAs in CRC tumor tissues and identified ZFAS1 (zinc finger antisense 1), previously observed to be tumor suppressor gene in human breast cancer [19, 20] and oncogene in hepatocellular carcinoma [21], to be up-regulated in CRC tissue. We further investigated whether ZFAS1 is detectable or altered in the tumor and non-tumor paired tissue of independent and larger cohort of CRC patients. We also evaluated the correlations between ZFAS1 expression levels in tumor tissues and clinico-pathological features of CRC. Finally, we examined whether ZFAS1 expression influences cell viability, cell cycle distribution, apoptosis and colony formation *in vitro* and which proteins and miRNAs have ability to interact with ZFAS1 and may participate on its functioning.

## RESULTS

### LncRNAs deregulated in CRC tissue

**Figure 1:**
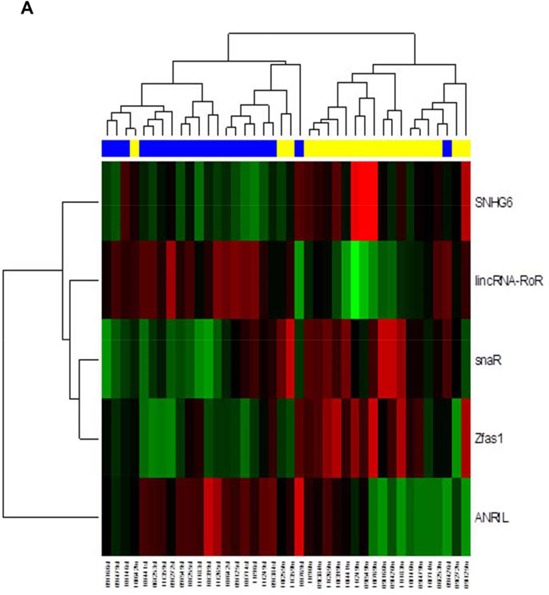

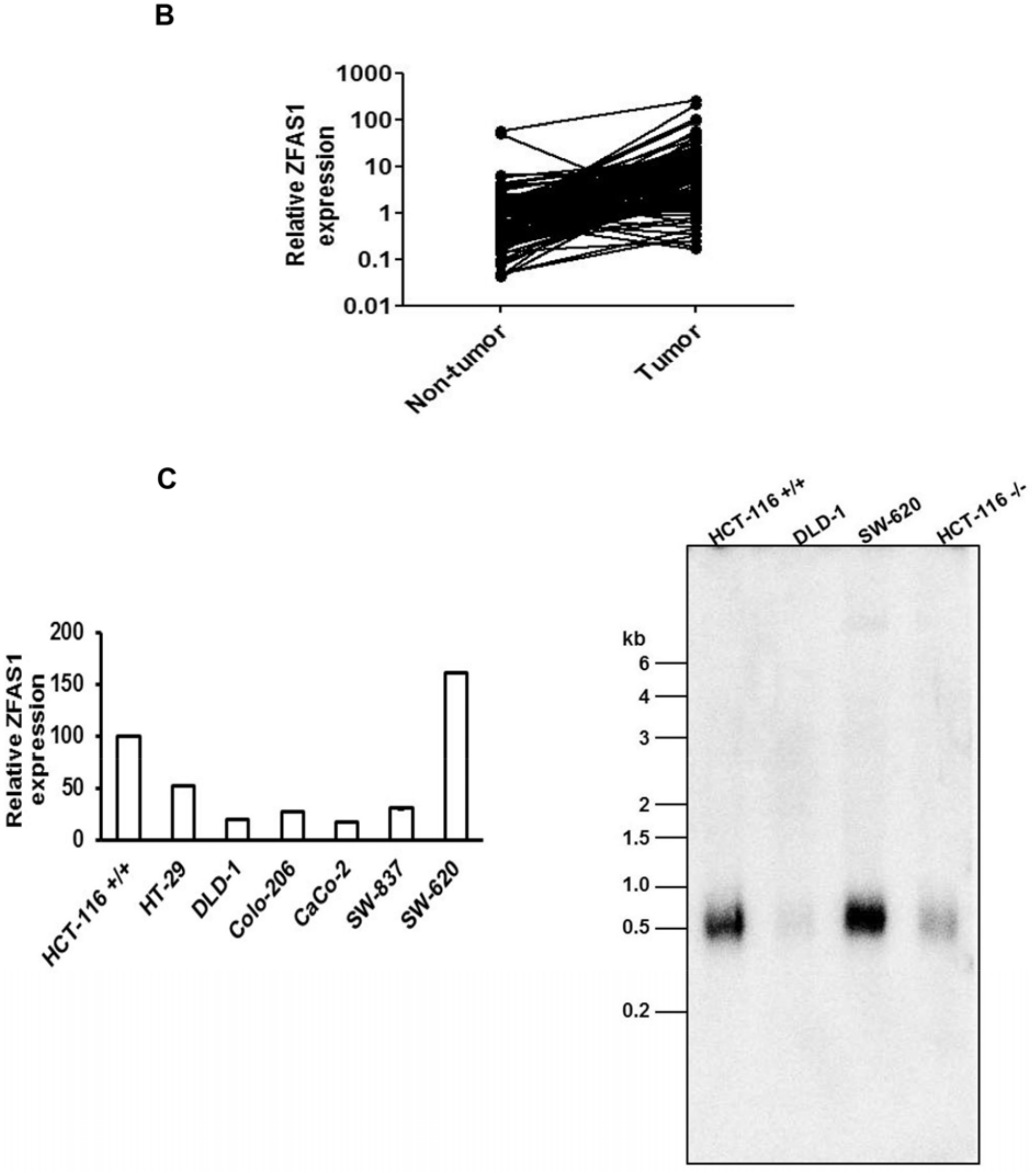

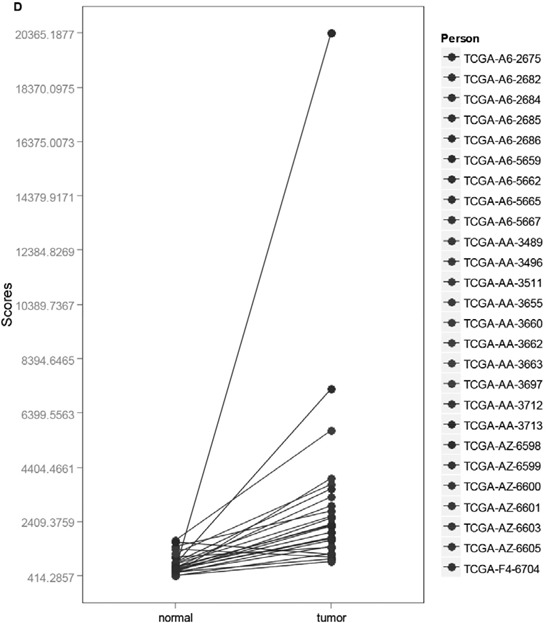
Analysis of ZFAS1 expression in CRC tissues and cell lines **A.** Hierarchical clustergram discriminating tumor and non-tumor tissue of CRC patients according to differentially expressed lncRNAs (yellow color indicates tumor samples of CRC patients, blue paired non-tumor colonic tissue, *P* < 0.01). **B.** Relative expression of ZFAS1 in CRC tissues (*n* = 119) compared with corresponding non-tumor tissues (*n* = 119). ZFAS1 expression was examined by real-time PCR. (*P* value < 0.001). **C.** The relative expression level of ZFAS1 in HCT116+/+, HT-29, DLD-1, Colo-206, CaCO-2, SW-837 and SW-620 cells was determined by real-time PCR. (C) The northern blot analysis to measure the ZFAS1 expression in CRC cells HCT116+/+, DLD-1, SW-620 and HCT116−/−. **D.** Based on the COADREAD, Illumina HiSeq-based TCGA dataset, expression levels of ZFAS1 are significantly higher in CRC tumor tissue in comparison to non-tumor colonic tissue (*P* < 10−11).

In the exploratory phase of the study we determined expression profiles of 83 lncRNAs, selected accordingly to their previous association with human pathology, in tumor and paired non-tumor colorectal tissues of 20 CRC patients (characterized as Exploratory cohort in Table 1). Further, we identified a signature of lncRNAs differentially expressed in CRC patients (6 up-regulated and 4 down-regulated; *P* < 0.01) (Figure 1A, Table 2). Based on the fold-change and level of significance we performed PubMed search for papers focused on five most deregulated lncRNAs (gene symbol for particular lncRNA was the only keyword used as a search strategy) and selected ZFAS1 for further independent validation as it was lncRNA being the least described.

**Table 1:** Patients characteristics

		Exploratory cohort *N* = 20	Validation cohort *N* = 119
Gender, *N* (%)	Male	11 (55)	72 (61)
	Female	9 (45)	47 (39)
Age at diagnosis	Median	70	68
	Range	48–87	40–85
BMI, kg/m^2^, *N* (%)	Normal (18–25)	7 (35)	42 (35)
	Overweight (26–30)	9 (45)	58 (49)
	Obese (< 30)	4 (10)	19 (16)
Smoker, *N* (%)	Non-smoker	12 (60)	68 (57)
	Short-term smoker	3 (15)	20 (17)
	Long-term smoker	5 (25)	31 (26)
TNM stage, *N* (%)	I	5 (20)	23 (19)
	II	5 (20)	39 (33)
	III	5 (20)	28 (24)
	IV	5 (20)	29 (24)
Grade, *N* (%)	1	8 (40)	31 (26)
	2	8 (40)	61 (51)
	3	4 (20)	26 (22)
	4	0	1 (0)
Tumor location, *N* (%)	Distal colon	13 (65)	63 (53)
	Proximal colon	7 (35)	56 (47)
Tumor size in diameter, *N* (%)	≤ 50 mm	17 (85)	104 (87)
	> 50 mm	3 (15)	15 (13)
Tumor invasion depth, *N* (%)	T1	2 (10)	1 (0)
	T2	6 (30)	27 (23)
	T3	10 (50)	78 (66)
	T4	2 (10)	13 (11)
Lymph node metastasis, *N* (%)	N0	10 (50)	66 (55)
	N1	7 (35)	30 (25)
	N2	3 (15)	23 (20)
Distant Metastasis, *N* (%)	M0	15 (60)	91 (76)
	M1	5 (40)	28 (24)

**Table 2:** Long non-coding RNAs differentially expressed between tumor and adjacent non-tumor tissues of CRC patients (*P* < 0.03)

Long non-coding RNA	Log Fold change	*P* Value	Adjusted *P* Value*
snaR	2.55	< 0.0001	0.0014
ANRIL	−2.81	< 0.0001	0.0017
lincRNA-RoR	−2.12	< 0.0001	0.0023
**ZFAS1**	**1.52**	**0.0004**	**0.0081**
SNHG6	1.11	0.0005	0.0081
Alpha 280	−1.88	0.0009	0.0123
lincRNA-VLDLR	−1.64	0.0010	0.0123
E2F4 antisense	−1.55	0.0016	0.0178
SCA8	1.07	0.0026	0.0248
lincRNA-SFMBT2	−1.49	0.0028	0.0248

* *P* value adjusted according to Bonferroni correction for multiple comparisons.

### ZFAS1 expression is increased in CRC tissue and cell lines

In the validation cohort of 119 CRC patients (characterized as Validation cohort in Table 1) we observed that 111 (93%) cases displayed at least two-times higher expression of ZFAS1 in CRC tissues in comparison to paired normal colorectal tissue (Figure 1B, *P* < 0.0001). ZFAS1 expression was determined also in CRC cell lines, including HCT116+/+ (p53 wild type), HCT116−/− (p53-null), HT-29, DLD-1(p53^241F^), Colo-206, CaCO-2, SW-837 and SW-620 cells. Our data indicated that ZFAS1 expression is significantly higher in HCT116+/+, SW-620, and HT-29 compared to other CRC cell lines (Figure 1B). Northern blot analysis further confirmed that ZFAS1 expression is significantly higher in HCT116+/+ and SW-620 in comparison to HCT116−/− and DLD-1 cells (Figure 1C). To evaluate ZFAS1 deregulation in CRC on expression data reached by different methodical approach we have downloaded and analyzed RNAseq TCGA-dataset COADREAD and confirmed significantly higher levels of ZFAS1 in CRC tumor tissue in comparison to non-tumor colorectal tissue (*P* < 10^−11^) (Figure 1D). We performed correlation analysis between ZFAS1 expression and various clinico-pathological features, and we have not observed any association of ZFAS1 expression with clinical stage, lymph node metastasis, distant metastasis, grade, tumor diameter and survival in our cohort (*P* > 0.05, data not shown).

### SiRNA selection for ZFAS1 silencing in CRC cells

The functional relevance of ZFAS1 in CRC cell lines was investigated by using ZFAS1 specific siRNAs. To silence ZFAS1 expression in CRC cells, three individual siRNAs were transiently transfected into the cell lines (Supplementary Figure S1A, 1B, and 1C). The silencing efficiency was evaluated in 24 hr to 120 hr after transfection using RT-qPCR in HCT116+/+, HCT116−/−, DLD-1, and SW-620 cells. Of the three siRNAs, n271359 siRNA demonstrated the highest silencing capacity (Supplementary Figure S1A). Thus, this siRNA was selected for ZFAS1 silencing in all subsequent experiments. We have not observed any effects of ZFAS1 silencing on SNORD12 levels (Supplementary Figure S2A) and also silencing of SNORD12 did not affect the expression of ZFAS1 (Supplementary Figure S2B).

### ZFAS1 silencing inhibits proliferation, cell cycle and colony formation of CRC cells

**Figure 2:**
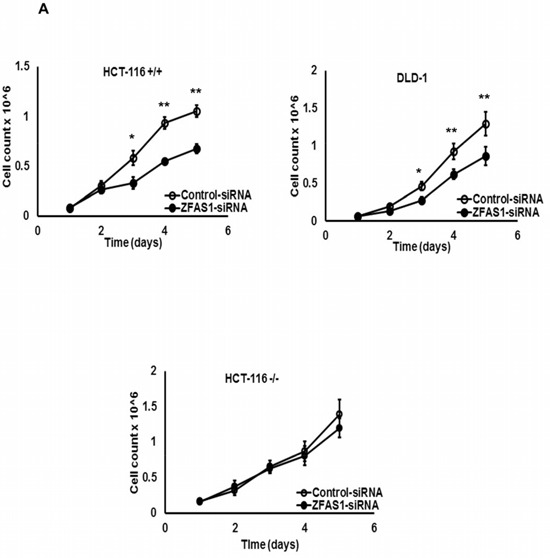

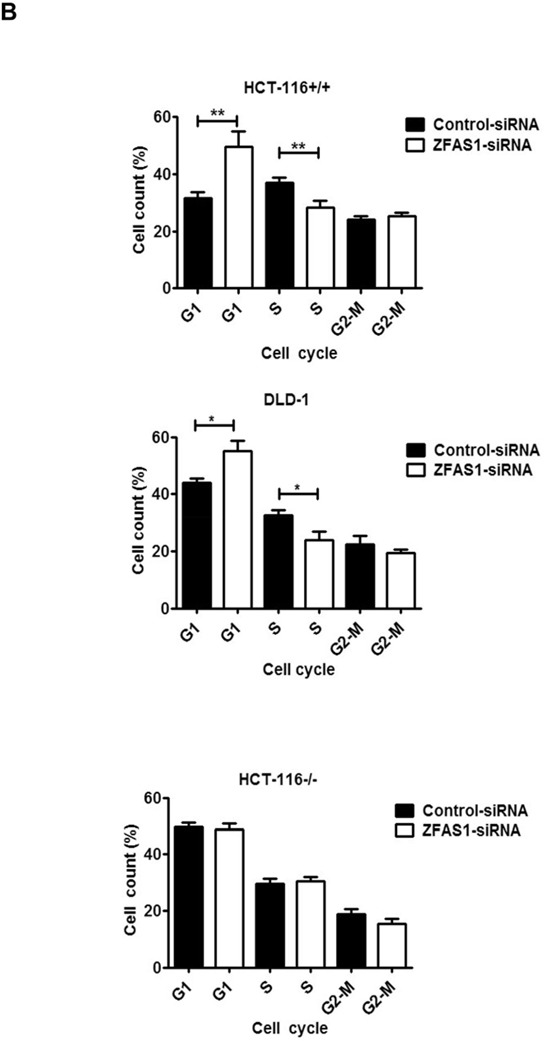

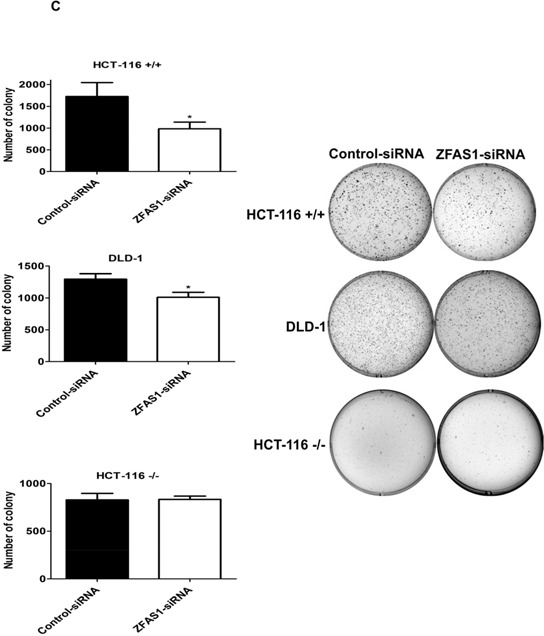
Effects of ZFAS1 knockdown on colorectal cancer cell proliferation *in vitro* **A.** Trypan blue exclusion method was performed to determine the proliferation of HCT116+/+, DLD-1, and HCT116−/− cells. Data represent the mean ± S.D. from three independent experiments. **B.** The effect of ZFAS1 silencing on cell cycle. The bar chart represents the percentage of cells in G0/G1, S, or G2/M phase, as indicated. **C.** Colony-forming growth assays were performed to determine the proliferation of HCT116+/+, DLD-1, and HCT116−/− cells after silencing of ZFAS1. The colonies were counted and captured. *P < 0.05, **P < 0.01.

ZFAS1 expression was silenced in HCT116+/+, HCT116−/− and DLD-1 cells. The down-regulation of ZFAS1 significantly inhibited the HCT116+/+ and DLD-1 cells proliferation (*P* < 0.05) (Figure 2A). However, there was no effect on cell proliferation observed in HCT116−/− cells (Figure 2A). Flow cytometry was used to assess whether ZFAS1 silencing in CRC cells is associated with changes in the distribution of cell cycle phases. As shown in Figure 2B, the percentage of CRC cells (HCT116+/+, DLD-1) in S-phase significantly decreased in cells with silenced ZFAS1 (*P* < 0.05). These results indicated that silencing of ZFAS1 expression lead to G1-arrest in CRC cells. To determine whether ZFAS1 expression silencing affects also tumorigenicity of CRC cells, colony formation assay was carried out. As shown in Figure 2C, silencing of ZFAS1 lead to significant decrease in number of colonies on soft agar in HCT116+/+ and DLD-1 cells (*P* < 0.05).

### ZFAS1 interacts with cyclin-dependent kinase 1

**Figure 3:**
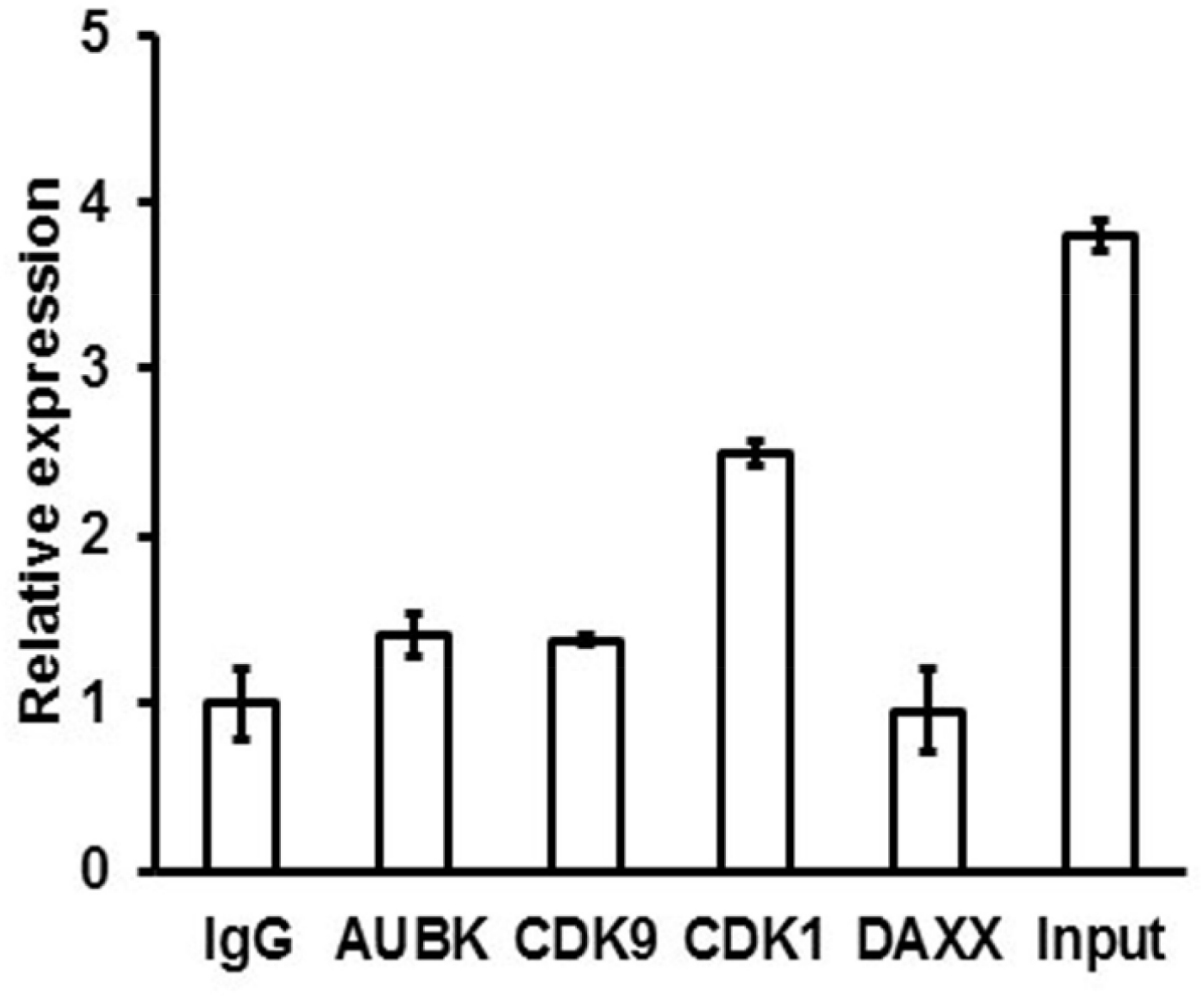
RNA-binding protein immunoprecipitation analysis was used to detect the interaction between ZFAS1 and Aurora kinase B, CDK9, CDK1 and DAXX As negative control IgG alone was used.

The ZFAS1 interacting proteins were identified by *in vitro* biotin–avidin pull-down system using protein lysates isolated from HCT116+/+ and DLD-1 cells (Supplementary Figure S3). The proteins identified by mass spectrometry analysis were selected based on the protein identification score and number of peptide matched (score > 100, peptide matched ≥ 5). The identified proteins were further narrowed down based on their function and previous association with cancer (Table 3). Based on GeneOntology analysis, most of these proteins were involved in the regulation of cell cycle checkpoint (fold enrichment > 5, *P* = 0,01) and cell cycle processes (fold enrichment > 5, *P* = 0,02). We selected Aurora kinase B (AUKB), Cyclin-dependent kinase 9 (CDK9), Cyclin-dependent kinase 1 (CDK1) and Death domain-associated protein 6 (DAXX) for further evaluation. Whether these candidate proteins can directly interact with ZFAS1 was evaluated by use of RNA-binding protein immunoprecipitation technique. Our experiments showed no interaction of ZFAS1 with DAXX, only little interaction with AUKB and CDK9, but significant interaction with CDK1 (Figure 3).

**Table 3:** Protein selected from *in vitro* pull down analysis

Accession	Protein	MW [kDa]	Scores	Peptides
CCAR2	Cell cycle and apoptosis regulator protein 2	102.8	255.8	7
CDC5L	Cell division cycle 5-like protein	92.2	940.8	19
DAXX*	Death domain-associated protein 6	81.3	638.7	15
MTA1	Metastasis-associated protein MTA1	80.7	136.9	5
ACINU	Apoptotic chromatin condensation inducer in the nucleus	151.8	134.6	5
CARF	CDKN2A-interacting protein	61.1	104.1	5
DKC1	H/ACA ribonucleoprotein complex subunit 4	57.6	713.2	14
TRI25	E3 ubiquitin/ISG15 ligase TRIM25	70.9	224.5	5
PRP4	U4/U6 small nuclear ribonucleoprotein Prp4	58.4	231.5	8
SSF1	Suppressor of SWI4 1 homolog	53.2	136	5
CAAP1	Caspase activity and apoptosis inhibitor 1	38.3	118.7	5
AURKB*	Aurora kinase B	39.3	293.6	7
CDK9*	Cyclin-dependent kinase 9	42.8	204.1	9
PCID2	PCI domain-containing protein 2	46.0	137.5	5
LYAR	Cell growth-regulating nucleolar protein	43.6	389.6	8
S30BP	SAP30-binding protein	33.8	223.4	5
PA2G4	Proliferation-associated protein 2G4	43.8	189.6	7
RUVB2	RuvB-like 2	51.1	263	7
MMTA2	Multiple myeloma tumor-associated protein 2	29.4	387.2	9
CDK1*	Cyclin-dependent kinase 1	34.1	159.1	5
CASPE	Caspase-14	27.7	103.2	6
RCD1	Cell differentiation protein RCD1 homolog	33.6	137.8	7
CD11A	Cyclin-dependent kinase 11A	33.6	137.8	8
THOC5	THO complex subunit 5 homolog	23.7	387.4	7
YBOX1	Nuclease-sensitive element-binding protein 1	35.9	495.7	9
RUVB1	RuvB-like 1	50.2	127.8	5
WDR5	WD repeat-containing protein 5	36.6	130.3	5
TNR1B	Tumor necrosis factor receptor superfamily member 1B	48.3	110.1	7

* Proteins selected for RNA-IP analysis.

### ZFAS1 is predicted to sponge miR-590-3p targeting cyclin-dependent kinase 1

By use of web-based prediction system we screened for potential ZFAS1 and miRNAs interactions and identified putative binding regions for miR-590-3p (1 target site, alignScore = 158) and miR-150-5p (1 target site, alignScore = 155) in ZFAS1 sequence. We evaluated potential of these miRNAs to target CDK1 and interestingly four algorithms used for prediction identified miR-590-3p to target 3′ untranslated region of CDK1. Consequently, we found in miRWalk database that miR-590-3p was experimentally proved to target CDK1 [22].

### ZFAS1 silencing lead to decrease in p53 and cyclin B1 levels and increased PARP cleavage

**Figure 4:**
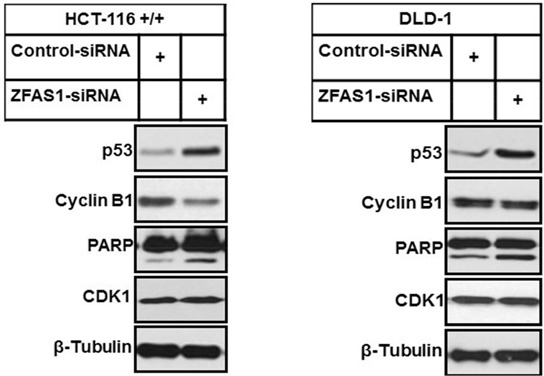
Western blot analysis of p53, Cyclin B1 and PARP cleavage after ZFAS1 siRNA transfection of HCT116+/+ and DLD-1 cells

We examined the levels of p53, CDK1, CDK1 partner cyclin B1 and PARP cleavage for detection of apoptosis after silencing of ZFAS1 in HCT116+/+ and DLD-1 cells. The western blot analysis indicated that expression levels of p53 were increased in both cell lines after ZFAS1 silencing. While CDK1 expression was not affected, the level of cyclin B1 was decreased in both cell lines after ZFAS1 silencing. Moreover, increase in PARP cleavage was observed in both cell lines with silenced ZFAS1 indicating higher apoptosis as a consequence of ZFAS1 silencing (Figure 4).

## DISCUSSION

By use of qPCR-based lncRNA profiling on the samples from the exploratory cohort of patients (*N* = 20) we have identified panel of 10 lncRNAs differentially expressed in CRC tissue and normal colorectal tissue (adjusted *P* < 0.03). We sorted these lncRNAs accordingly to the fold-change and level of significance and performed PubMed search to identify which of these lncRNAs is the least discovered. Based on that ZFAS1 was selected for further independent validation. Then we validated ZFAS1 on independent cohort of patients and observed very high frequency (more than 90%) of at least two-times higher expression levels of ZFAS1 in human CRC tissues when compared to normal colorectal tissue suggesting that ZFAS1 may play an important role in CRC pathogenesis. We tried to correlate ZFAS1 levels to clinico-pathological features of CRC patients including survival and did not observe any significant associations indicating ZFAS1 deregulation to be one of the common and early events in CRC carcinogenesis.

When compared to two studies focused on ZFAS1 in breast cancer, we reached contradictory data. The first study published in 2011 described mainly structural features of ZFAS1 gene and transcript on mouse model [15]. Authors focused on breast cancer and performed several experiments also with human breast cancer cell lines and breast cancer tumors and non-tumor adjacent tissues. They revealed contrasting observations to our results (decreased levels of ZFAS1 in tumor tissue, increase in cell proliferation after ZFAS1 silencing), and considered ZFAS1 as tumor suppressor gene in breast cancer. However, the number of tumor tissue samples evaluated in this study was very low (only 5) and effects on the proliferation were evaluated only in one time point (48 h) and without biological replicates [19]. Moreover, discrepancies between our results and previous ZFAS1 study could be explained also by differences in pathology of colorectal and hormonal-dependent breast cancer. Another group focused in ZFAS1 levels in invasive breast carcinoma, ductal carcinoma *in situ* and normal adjacent breast tissues by use of chromogenic *in situ* hybridization and human FFPE tissues [20]. In this study ZFAS1 was negative or weakly expressed among all groups of samples indicating there is no significance of ZFAS1 in breast cancer.

Concordantly with our results, in hepatocellular carcinoma (HCC) ZFAS1 was identified as one of the most frequently amplified genes based on the publicly available microarray data [21]. ZFAS1 was described to function as an oncogene involved in metastatic progression of HCC and authors suggest that this function is associated with ZFAS1 sponging activity on miR-150, which is known to be tumor suppressor in HCC [21]. Contrary to our results ZFAS1 indicated prognostic potential in HCC.

To further discover functioning of ZFAS1 in CRC we established siRNA enabling efficient silencing of ZFAS1 and evaluation of its effects on CRC cells proliferation, cell cycle and tumorigenicity. We observed that ZFAS1 silencing lead to significant inhibition of CRC cells proliferation, probably through to G1-arrest, and decrease in CRC cells tumorigenicity. These observations are in disagreement with results of the study in HCC, where the main cellular effects caused by ZFAS1 are associated to invasion and metastatic potential [21].

Interestingly, ZFAS1 is known to host three C/D box-containing homologous snoRNA genes, SNORD12, SNORD12b, and SNORD12c [15]. To confirm that that cellular effects observed after ZFAS1 silencing in CRC cells are not a consequence of reduced SNORD12 expression, we evaluated effects of ZFAS1 silencing on SNORD12 levels and found that there is just a minor change in SNORD12 expression in ZFAS1-silenced cells (Supplementary Figure S2A). These minor changes in SNORD12 expression, when compared to the ~90% silencing of the host transcript, suggest that the cellular effects observed following ZFAS1 silencing are a consequence of ZFAS1 mature transcript functioning. Furthermore, silencing of SNORD12 expression did not affect the expression of ZFAS1 (Supplementary Figure S2B).

Another aim of our study was to identify protein interaction partners of ZFAS1 and discover mechanism responsible for this cell cycle-based oncogenic functioning. The ZFAS1 interacting proteins were identified by RNA pull-down assay with subsequent protein detection by mass spectrometry. Based on that AUKB, CDK9, CDK1 and DAXX were further evaluated by RNA-binding protein immunoprecipitation, whereas significant interaction was proved only in case of CDK1. To further investigate how ZFAS1 down-regulation induces CRC cells growth arrest and apoptosis, we examined the levels of p53, CDK1, CDK1 partner cyclin B1 and PARP cleavage for detection of apoptosis after silencing of ZFAS1 in HCT116+/+ and DLD-1 cells. After ZFAS1 silencing protein levels of p53 significantly increased. While CDK1 levels were not affected, the levels of cyclin B1 decreased in both cell lines (Figure 4). Moreover, increase in PARP cleavage was observed indicating induction of apoptosis as consequence of ZFAS1 silencing.

ZFAS1 functioning to destabilize p53 is supported by the fact, that inhibition of proliferation, cell cycle arrest and induction of apoptosis were observed exclusively in cell lines with wild-type p53 (HCT116+/+) or with p53 having ability to be functionally restored (DLD-1), whereas in the first mentioned the effects were more prominent. On other hand, there was no effect observed in p53-null cell line (HCT116−/−). ZFAS1-destabilizing effect on p53 is further supported by the fact, that ZFAS1 silencing led to induction of p53 in both examined cell lines. Therefore, our observations are indicative for involvement of ZFAS1 in p53-dependent regulatory pathways in cancer cell but without clear explanation of the mechanism at the moment.

As mentioned above we observed significant G1-arrest as a consequence of ZFAS1 silencing in CRC cells. Our immunoprecipitation experiment confirmed interaction of ZFAS1 with CDK1 and our consequent analysis confirmed decrease of CDK1 partner cyclin B1 after ZFAS1 silencing with CDK1 being not affected. These data indicate involvement of ZFAS1 in regulation of cyclin B1 and as a consequence CDK1/cyclin B1 complex and cell cycle progression. This mechanism is well known to be involved in G2 cells progression into M phase, and our concurrent observation associated with ZFAS1 silencing is G1-arrest of cell cycle. However, the participation of CDK1 on stimulation of G1/S transition was also described. CDK1 activity at the G1/S transition may have previously escaped detection due to the fact that it appeared negligible compared with the maximal activity peak in G2/M [23]. Nevertheless, low levels of CDK1 activity are sufficient to drive cells into S phase and initiate DNA replication in the absence of CDK2. In the presence of CDK2, CDK1 may still be the predominant kinase at G1/S, whereas CDK2 may have modulatory function. Alternatively, CDK1 and CDK2, and probably other CDKs, may act synergistically or redundantly to promote the G1/S transition [23]. Therefore, we hypothesize, that ZFAS1 facilitate G1/S transition in CRC cells through direct interaction with CDK1 and regulation of cyclin B1 levels.

Moreover, we have found another link between ZFAS1 and CDK1 based on ZFAS1-miRNA interaction. By use of web-based prediction system we have identified putative binding regions for miR-590-3p and miR-150-5p in ZFAS1 sequence. MiR-150-5p was already described and experimentally proved to be sponged by ZFAS1 in HCC and associated with tumor invasion and metastasis [21]. We focused on miR-590-3p and found experimental evidence showing miR-590-3p to directly target CDK1 [22]. It seems that while in HCC ZFAS1 sponging activity on miR-150-5p influence mainly metastatic potential, in CRC ZFAS1 sponges miR-590-3p and trough CDK1 affects cell cycle and proliferation.

**Figure 5:**
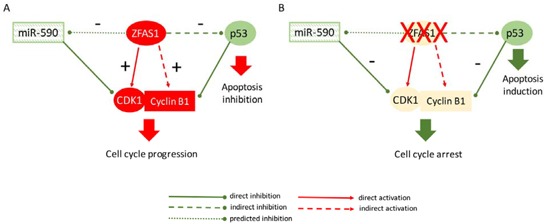
Proposed model of ZFAS1 functioning in cell cycle control and apoptosis in CRC **A.** Up-regulated ZFAS1 acts as oncogene in CRC via destabilization of p53 and through interaction with CDK1/cyclin B complex leading to cell cycle progression and inhibition of apoptosis. **B.** Silencing of ZFAS1 lead to accumulation of p53, attenuation of CDK1/cyclin B complex and finally cell cycle arrest and induction of apoptosis in CRC cells. ZFAS1 is predicted to sponge miR-590 targeting CDK1.

In conclusion, our data indicate that ZFAS1 functions as an oncogene in CRC by two main actions: (i) via indirect destabilization of p53 and through (ii) direct and indirect interactions with CDK1/cyclin B complex leading to cell cycle progression and inhibition of apoptosis (Figure 5). However, molecular mechanisms behind these interactions have to be further clarified.

## MATERIALS AND METHODS

### Patient and tissue sample

One hundred and nineteen CRC patients who underwent surgery at Masaryk Memorial Cancer Institute (Brno, Czech Republic) between 2008 and 2011 were included in this study. The data on all subjects were obtained from medical records and pathology reports. The data collected included age, gender, smoking habits, BMI, disease-free survival overall survival and tumor features such as tumor size, clinical stage, tumor invasion depth, tumor location, and occurrence of distant metastasis (summarized in Table 1). All subjects were of the same ethnicity (Caucasian). The study has been approved by the local ethical committee. Tumor and adjacent normal tissues were snap-frozen in liquid nitrogen immediately after extraction and stored at −80°C until total RNA was extracted.

### RNA extraction

Total RNA was extracted from tissues and cells using mirVana miRNA Isolation Kit (Ambion, Austin, TX, USA) according to the manufacturer's instruction. Concentration and purity of RNA were determined spectrophotometrically by measuring its optical denstity (A260/280 > 2.0; A260/230 > 1.8) using a NanoDrop ND-1000 (Thermo Scientific, Wilmington, DE, USA).

### LncRNA profiling

To focus on those clinically relevant lncRNAs, in exploratory phase of the study, 83 candidate lncRNAs were determined by lncRNA profile qPCR arrays (System Biosciences, Mountain view, CA). Analysis of the RT-qPCR data was performed using SDS version 2.0.1 software (Applied Biosystem, Foster City, CA, USA). RNU43 has been chosen as reference gene for normalization of lncRNAs expression levels. The relative expression levels of target lncRNAs were determined by the equation 2^−ΔCT^, in which Δ*C*_T_ were calculated as follows: Δ*C*_T_ = *C*_TlnRNA-of-interest_ – *C*_TRNU43_. Relative lncRNA levels were then calculated with the RQ Manager 1.2. Normalized expression data from profiling phase of the study were statistically evaluated.

### Reverse transcription (RT) and quantitative PCR (qPCR)

After RNA extraction High-capacity cDNA reverse transcription kit (Applied Biosystem, Foster City, CA, USA), was used to synthesize cDNA according to the manufacturer's recommendations. The expression levels of ZFAS1 were detected by RT-qPCR using Taqman non-coding RNA assay and Taqman gene expression master mix (Applied Biosystem, Foster City, CA, USA). PCR was performed using the Applied Biosystem 7500 Sequence Detection System.

### Cell lines and culture condition

Human CRC cell lines, including HCT116+/+ (p53 wild type), HCT116−/− (p53 knockout), HT-29, DLD-1(p53^241F^), Colo-206, CaCO-2, SW-837 and SW-620 were obtained from the American Type Culture Collection (USA), and were maintained in Dulbecco's Modified Eagle Medium supplemented with 10% FBS, 100 μg ml^−1^ penicillin, 100 μg ml^−1^ streptomycin, 0.1 mM nonessential amino acids, 2 mM L-glutamin, and 1 mM sodium pyruvate (Invitrogen, Gibco, Carlsbad, CA, USA) in 5% CO_2_ at 37°C.

### Northern blot analysis

Total RNA from CRC cells was purified. A ZFAS1-specific, radioactive DNA probe with a length of 300 bp was generated using [α-^32^P]dCTP (Perkin Elmer) and the Megaprime DNA labelling system (GE Healthcare, Pittsburgh, PA, USA). Hybridization was performed using QuickHyb (Agilent, Santa Clara, CA, USA), following the manufacturer's instructions.

### Transfection of colorectal cancer cells

Pre-designed silencer select small interference RNA (siRNA) specific to ZFAS1 (siRNA ID n271359, and n271357 – A, B), and the control siRNA was obtained from Applied Biosystem (Foster City, CA, USA). The custom synthesized ZFAS1 siRNA (sense 5′-CUGGCUGAACCAGUUCCACAAGGUU-3′, and the corresponding antisense RNA) and SNORD12 siRNA (sense 5′-CUGUUGAUCUCUACACUAUtt-3′ and the corresponding antisense RNA) were obtained from IDT (Coralville, Iowa, USA). siRNA oligonucleotides were transfected in to (HCT116+/+ [p53 wild type], HCT116−/− [p53 knockout], DLD-1 (p53^241F^) cells using Lipofectamine RNAiMAX transfection reagent accordingly to manufacturer's instructions (Invitrogen).

### Cell viability assay

Cell viability was measured by Trypan blue exclusion method. The colorectal cancer cells (HCT116+/+ (p53 wild type), HCT116−/− (p53 knockout), DLD-1 (p53^241F^) were transfected with ZFAS1 siRNA and control siRNA and evaluated in different time point (24 hr to 120 hrs), the transfected cells were harvested and the cell suspensions were mixed with 0.4% of trypan blue solution and viable cells were counted by using hemocytometer. All measurements were repeated three times in quadruplicates.

### Cell cycle analysis

CRC cells (HCT116+/+ (p53 wild type), HCT116−/− (p53 knockout), and DLD-1(p53^241F^)) were transfected with ZFAS1 siRNA. The transfected cells were harvested by trypnization and fixed with 70% ethanol. Subsequently, the cells were washed in PBS and treated with 0.1 mg ml^−1^ RNase for 30 min at 37°C. Finally, 1 mg ml^−1^ propidium iodide was added and another 10 min of incubation at room temperature. The percentages of cells in various phases of cell cycle were determined using FACS Canto II (BD Biosciences, San Jose, CA) and analyzed by FlowJo 7.2.2. (Tree Star, Ashland, OR). All measurements were repeated three times in triplicates.

### Soft agar colony formation assay

Cells were transfected with the ZFAS1 siRNA or control siRNA. Forty-eight hours after transfection, cells were trypsinized, and 5 × 10^3^ cells were mixed with a 0.35% agar solution in DMEM media containing 10% FBS and layered on top of a 0.75% agar layer in six-well tissue culture plates. The plates were incubated for 1–2 weeks at 37°C in a 5% CO_2_ atmosphere until colonies were formed. The colonies were counted by using Gel count (Oxford Optronix Ltd, Abingdon UK). All measurements were repeated three times in triplicates.

### RNA pull-down assay with subsequent protein detection

For *in vitro* RNA pull-down, template ZFAS1 DNA was prepared by PCR using specific primers (5′-CTTTCGCGTCTGCGGTGCCCGG and 3′-GCAG GTAGGCAGTTAGAAATTTC). The PCR product were ligated in to pGEM-T Easy Vector, and transformed in to competent cells. 5 μg of prepared plasmid was digested and purified with QIAquick PCR clean-up Kit (Qiagen). ZFAS1 was labelled with biotin-UTP (Roche) during *in vitro* transcription reaction. The 25 μl of biotinylated RNA was incubated over night with 3 mg protein lysate and 30 μl of MyOne Streptavidin C1 Dynabeads (Invitrogen), at 4°C with rotation. After several washes magnetic beads were resuspended in 15 μl protein-loading buffer, RNA-bound protein separated by SDS-PAGE and visualized by Coomassie G-250. The unique protein bands were excised from gel and in-gel digested with trypsin, detected by mass spectrometry analyses.

### RNA-binding protein immunoprecipitation

RNA-binding protein immunoprecipitation (RIP) was performed according to the manufacturer's protocols from the EZ-Magna RIP Kit (Millipore, USA). Briefly, HCT-116^+/+^ cells were rinsed with ice-cold phosphate buffered saline (PBS), lysed by RIP lysis buffer. The Aurora kinase B (#3094), CDK9 (#2316), CDK1 (#9116) antibodies (Cell Signaling Technology, Danvers, MA) and DAXX (#07–471) antibody (Upstate, Lake Placid, NY, USA) and nonspecific control normal IgG antibodies were used for the immunoprecipitation. RIP lysates and magnetic beads-bound antibodies were incubated together with rotating for overnight at 4°C. Afterwards, proteins in the immunoprecipitate were digested with proteinase K and bound RNAs were purified from the supernatants, The RNA concentration was measured by a NanoDrop (Thermo Scientific, Wilmington, DE, USA). Furthermore, purified RNA was subjected to RT-qPCR analysis to demonstrate the presence of the binding target.

### Western blot assay and antibodies

Cell protein lysates were separated by sodium dodecyl sulfate-polyacrylamide gel electrophoresis (SDS-PAGE), transferred to PVDF membranes, and subjected to Western blot analysis utilizing various antibodies. The antibodies utilized were obtained from the following sources: p53 (#2527), PARP (#9532), total CDK1 (#9116), cyclin B1 (#12231), and β-tubulin, (#2128) (Cell Signaling Technology, Danvers, MA).

### Prediction of miRNAs sponged by ZFAS1 and these miRNAs targets

StarBase v 2.0 (http://starbase.sysu.edu.cn/) was developed by analyzing a large set of Ago and RBP binding sites derived from all available CLIP-Seq experimental techniques (PAR-CLIP, HITS-CLIP, iCLIP, CLASH), and has shown extensive and complex RNA–RNA and protein–RNA interaction networks [24]. We have used this web-based application to discover potential ZFAS1-microRNAs interactions. The target genes of differentially expressed miRNAs were predicted by 4 bioinformatic algorithms (miRanda, miRWalk, RNA22 and Targetscan) by the online tools of miRWalk (http://www.umm.uni-heidelberg.de/apps/zmf/mirwalk/) [25].

### Statistical analysis

Expression data from lncRNAs profiling were statistically evaluated in the environment of statistical language R by use of Bioconductor package and LIMMA approach combined with hierarchical clustering (HCL). *P values were adjusted according to Bonferroni correction for multiple comparisons. The data from *in vitro* experiments are presented as the mean values ± SD. Statistical differences between ZFAS1 expression levels in CRC patients and healthy controls and different subgroups of CRC patients defined by various clinico-pathological features were evaluated by non-parametric Mann-Whitney U test. Cut-off value enabling to divide patients into ZFAS1 low- and high-level group for purpose of survival analysis was determined using Receiver Operator Characteristic (ROC) analysis. The survival analyses were performed using Kaplan-Meier plots approach. All calculations were performed using GraphPad prism software version 5.0. *P* values of less than 0.05 were considered statistically significant.

## SUPPLEMENTARY FIGURES


