# Supplementary material for: Long non-coding RNA ZFAS1 interacts with CDK1 and is involved in p53-dependent cell cycle control and apoptosis in colorectal cancer
Source: Oncotarget. 2015 Oct 19;7(1):622–37. doi: 10.18632/oncotarget.5807 (PMC4808022; doi:10.18632/oncotarget.5807)
Supplement: Supplementary file 1 [file oncotarget-07-0622-s001.pdf]

SUPPLEMENTARY FIGURES

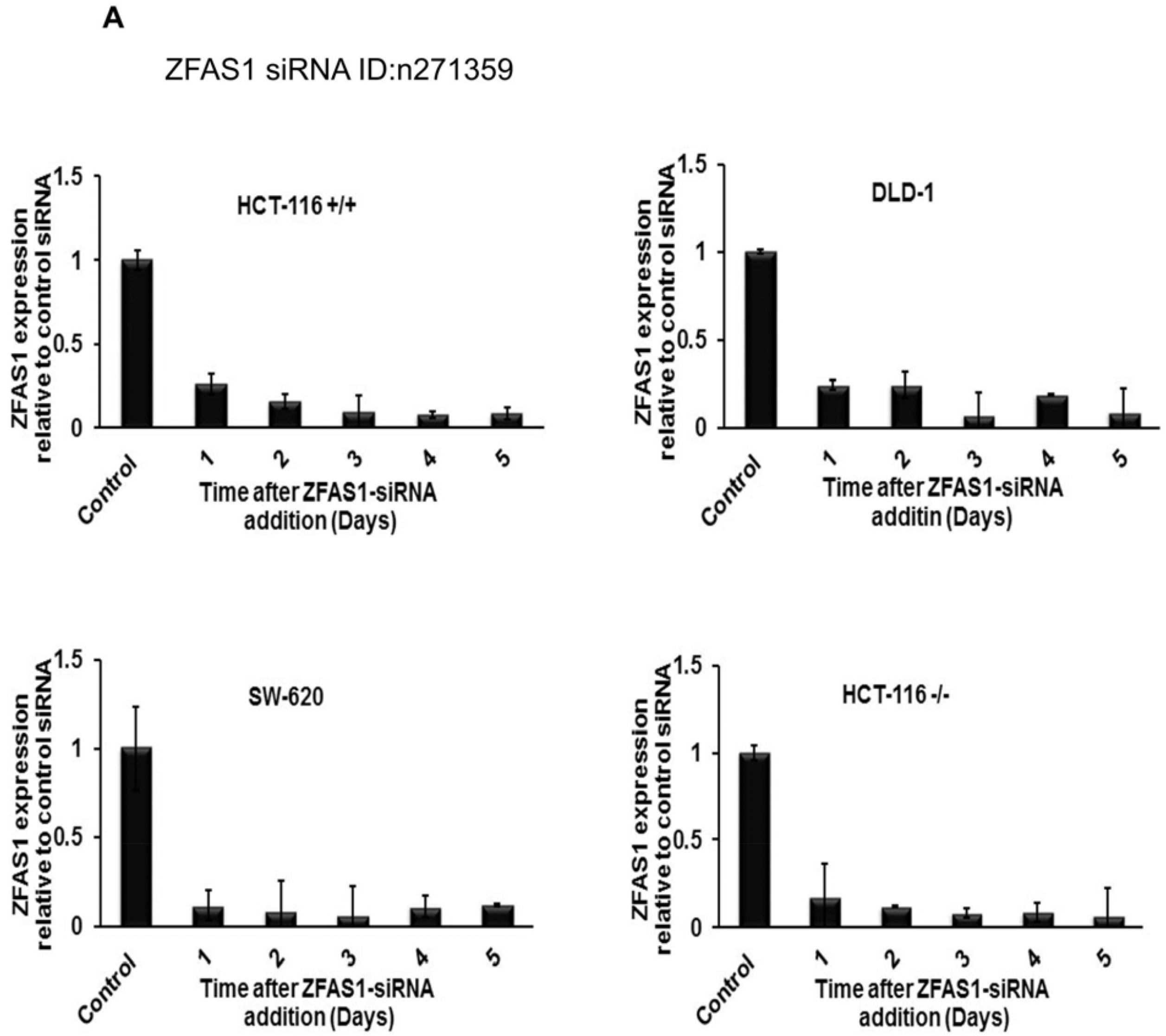

Supplementary Figure S1: Selection of most efficient siRNA for ZFAS1 silencing. The CRC cells HCT116+/+, DLD-1, SW-620 and HCT116−/− were transfected with ZFAS1 specific siRNA (n271359, n271357 and 5′-CUGGCUGAACCAGUUCCACAAGGUU-3′) and their silencing efficiencies were evaluated by determination of ZFAS1 expression levels. (*Continued*)

B

ZFAS1 siRNA ID: n271357

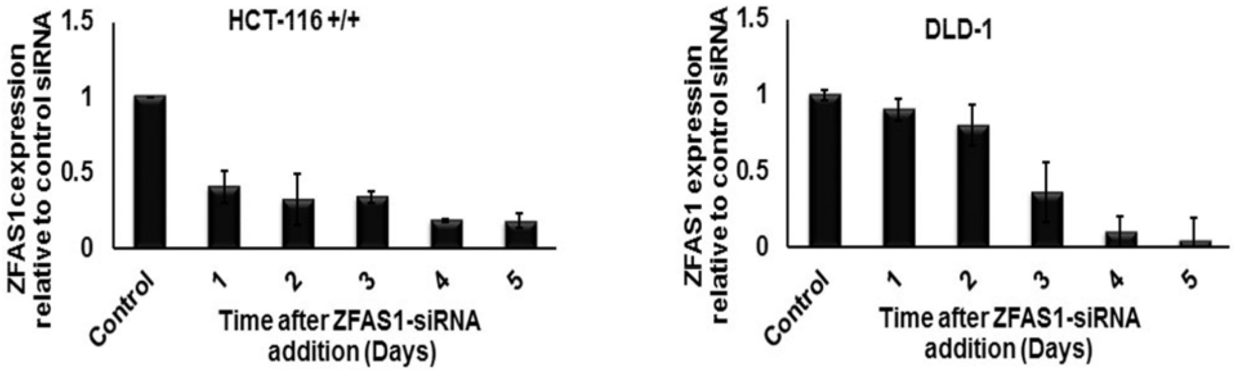

C

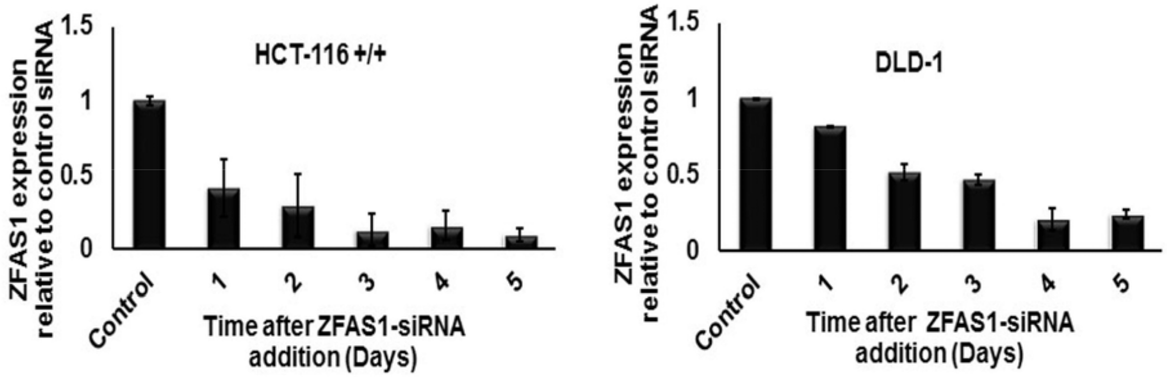

Supplementary Figure S1: (*Continued*) Selection of most efficient siRNA for ZFAS1 silencing. The CRC cells HCT116+/+, DLD-1, SW-620 and HCT116−/− were transfected with ZFAS1 specific siRNA (n271359, n271357 and 5′-CUGGCUGAACCAGUU CCACAAGGUU-3′) and their silencing efficiencies were evaluated by determination of ZFAS1 expression levels.

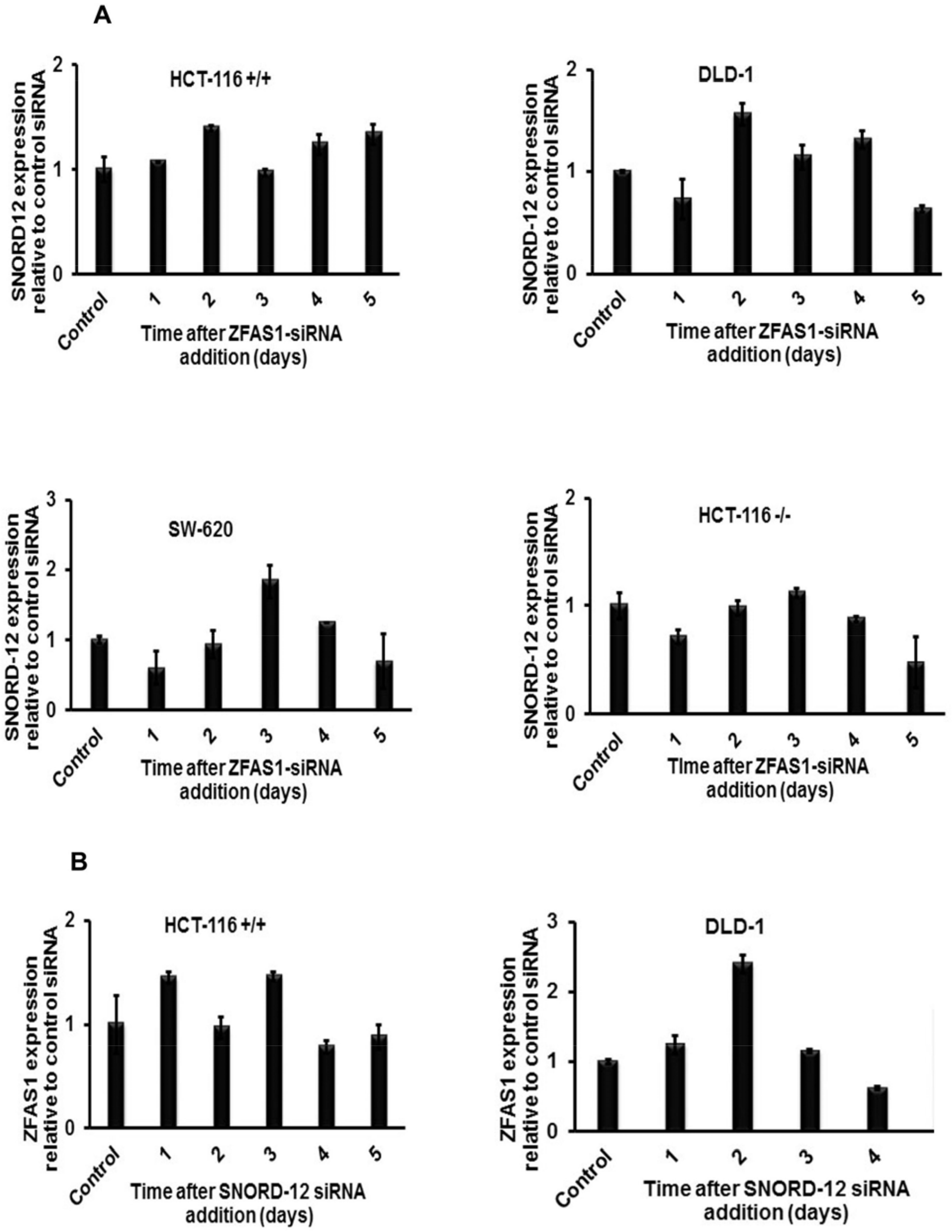

Supplementary Figure S2: Expression of SNORD12 in CRC cells after ZFAS1 silencing. A. The expression of ZFAS1 in CRC cells was silenced by using ZFAS1 specific siRNA. The level of SNORD12 was measured in these cell lines to check whether ZFAS1 knockdown affects the SNORD12 expression. **B.** The expression of SNORD12 in colorectal cancer cells was silenced by using SNORD12 specific siRNA. The expression level of ZFAS1 was measured in these cell lines to check whether SNORD12 silencing affects the ZFAS1 expression.

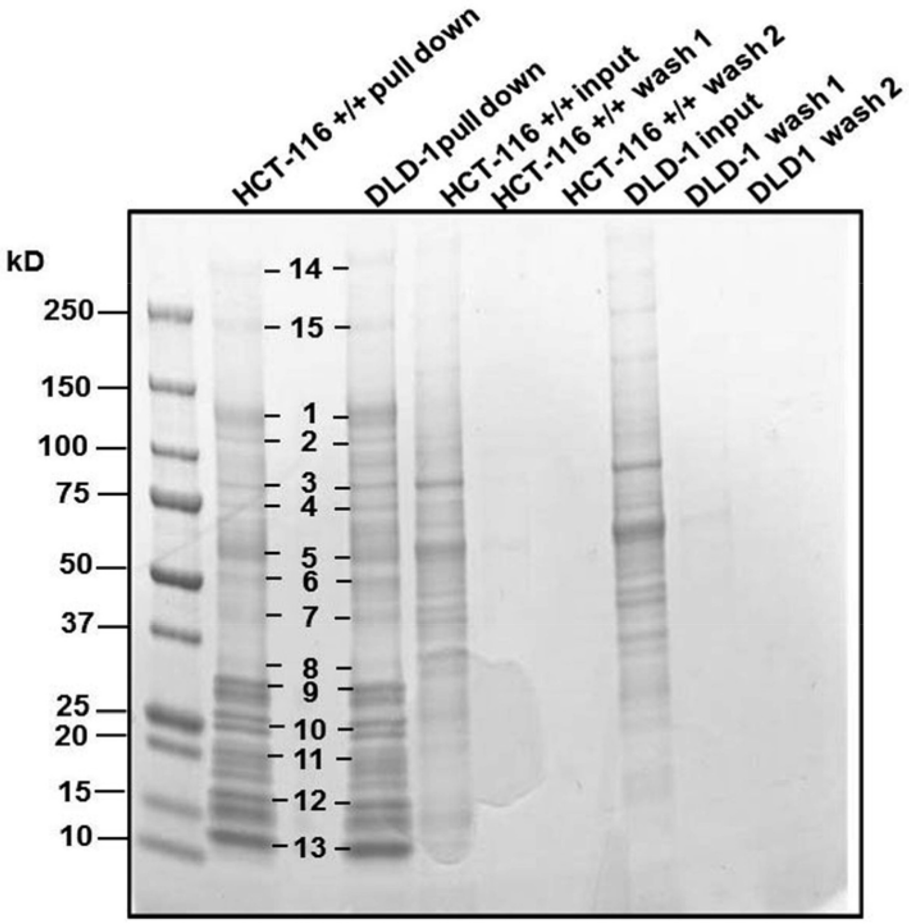

Supplementary Figure S3: *In vitro* RNA pull-down was performed using ZFAS1 template and RNA-bound protein separated by SDS-PAGE. The protein bands were excised and detected by mass spectrometry analysis.
